# MitoBlue as a tool to analyze the mitochondria-lysosome communication

**DOI:** 10.1038/s41598-020-60573-7

**Published:** 2020-02-26

**Authors:** Mateo I. Sánchez, Yolanda Vida, Ezequiel Pérez-Inestrosa, José L. Mascareñas, M. Eugenio Vázquez, Ayumu Sugiura, José Martínez-Costas

**Affiliations:** 1 Centro Singular de Investigación en Química Biolóxica e Materiais Moleculares (CiQUS), Departamento de Química Orgánica, Universidade de Santiago de Compostela, 15782 Santiago de, Compostela Spain; 2 Montreal Neurological Institute, McGill University, Montreal, QC Canada; 3 Department of Neurology and Neurosurgery, McGill University, Montreal, QC Canada; 4 Centro Andaluz de Nanomedicina y Biotecnología-BIONAND. Parque Tecnológico de Andalucía, c/Severo Ochoa, 35, 29590 Campanillas, Málaga Spain; 5 Centro Singular de Investigación en Química Biolóxica e Materiais Moleculares (CiQUS), Departamento de Bioquímica y Biología Molecular, Universidade de Santiago de Compostela, 15782 Santiago de, Compostela Spain; 6 Universidad de Málaga-IBIMA, Departamento de Química Orgánica. Campus de Teatinos s/n, 29071 Málaga, Spain

**Keywords:** Imaging, Mitochondria, Membranes

## Abstract

MitoBlue is a fluorescent bisamidine that can be used to easily monitor the changes in mitochondrial degradation processes in different cells and cellular conditions. MitoBlue staining pattern is exceptional among mitochondrial dyes and recombinant fluorescent probes, allowing the dynamic study of mitochondrial recycling in a variety of situations in living cells. MitoBlue is a unique tool for the study of these processes that will allow the detailed characterization of communication between mitochondria and lysosomes.

## Introduction

Cells maintain a functional mitochondrial network and avoid the buildup of defective mitochondria through a complex equilibrium between organelle fission and fusion, which isolates damaged organelles and redistributes their contents for recycling^1–3^. Mitochondrial quality control is maintained through multiple pathways, including intramitochondrial proteases, proteasomal degradation, mitochondrial-derived vesicles (MDVs), and mitophagy—the specific autophagic degradation of mitochondria^4–6^. Additionally, functional mitochondria are dependent on a number of dynamic processes, including fusion, fission, mobility, and direct contact with other organelles. Thus, mitochondrial quality control and dynamics are tightly coupled, and the disruption of this equilibrium leads to abnormal dynamics^4^. Disorders in mitochondrial homeostasis is at the origin of many physiopathological conditions and diseases^7^, including cancer or neurodegenerative diseases, such as Alzheimer’s and Parkinson’s disease^8,9^. Furthermore, mitochondrial quality control seems to have a close link with aging^10–12^. Thus, given its central role in such a wide array of fundamental processes^13^, the development of probes to study mitochondria dynamic processes represents an important challenge with great potential in basic and applied biomedical research^14–16^.

Mitochondrial quality can be evaluated by imaging analysis with fluorescent microscopy, using antibodies, reporter proteins or chemical probes, and by electron microscopy (EM) to characterize mitochondrial ultrastructures^17–19^. However, most of these techniques require cellular fixation, which can affect cellular structures and prevent the observation of dynamic processes, such as the formation of vesicles involved in some of the alternative degradation mechanisms affecting mitochondria. Recent imaging techniques allow the visualization of mitochondria associated molecular events with extraordinary sensitivity, and with selectivity and spatio-temporal resolution^20–22^. For example, using the reporter mRFP-GFP-LC3 it is possible to label different stages in autophagy by monitoring the evolution of the fluorescence emission profile resulting from the acidification of the autophagosome^23,24^. However, despite their widespread use, biosensors based on large protein constructs suffer from significant limitations, such as the need for transfection and over-expression, relatively low photostability, and large size that can lead to interference at the molecular level, poor biodistribution and pharmacokinetics, or immune response^25^. Small-molecule fluorophores offer several advantages over genetically encoded probes, such as highly efficient and homogeneous staining and bright emission. Furthermore, given that they do not need to be expressed, they can be used in cell cultures by simple incubation protocols^26^. In addition to classic Mitotracker stains that localize in the mitochondria^27,28^, a number of alternative mitochondrial dyes have been recently described^29–34^. Indeed, to the best of our knowledge, there is only one example in the literature of an organic probe that can be used to monitor mitophagy. This compound consists of a cyanine dye that stains mitochondria (emitting at 650 nm), and that upon mitophagic degradation is transported into the acidic lysosomes, where in its protonated form emits at a longer wavelength (750 nm)^35^.

**Figure 1:**
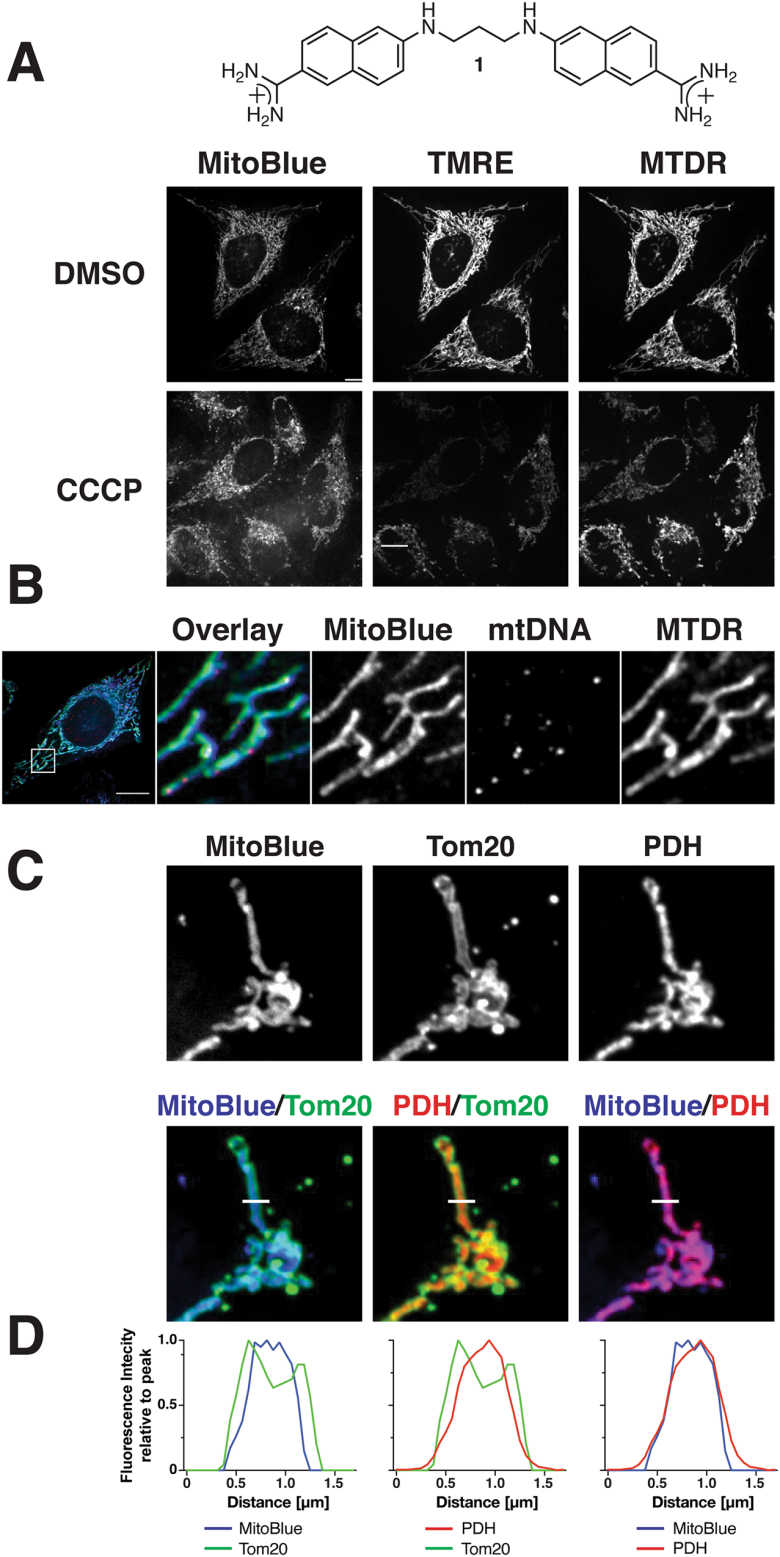
Mitochondrial localization of MitoBlue (1). (**A**) MitoBlue is a membrane potential-independent dye. HeLa cells were incubated with 5 µM MitoBlue, 200 nM tetramethylrhodamine ethyl ester (TMRE), and 50 nM Mitotracker Deep Red (MTDR) after five min preincubation with either DMSO (top panels) or 100 µM CCCP (bottom panels). Scale bars: 20 μm. (**B**) No binding to mtDNA *in cellulo*. HeLa cells were incubated with 5 µM MitoBlue and 100 nM Mitotracker Deep Red (MTDR) in standard conditions, washed, and further incubated in culture medium for 15 min at 37 °C. The cells were fixed and subjected to immunofluorescence staining using anti-DNA antibody. Scale bar: 10 μm. (**C**) MitoBlue intra-mitochondrial localization. COS-7 cells labeled with 5 μM MitoBlue were subjected to immunofluorescence staining using anti-Tom20 and -PDH antibodies. Scale bar: 5 μm. (**D**) Line scan co-localization analysis of MitoBlue, PDH, and Tom20, corresponding to the white line on the left image in (**C**).

We have recently reported the synthesis and biophysical characterization of MitoBlue (**1**, Fig. 1), a blue-emitting fluorescent dye that stains functional mitochondria, displaying low toxicity and high resistance to photobleaching, even after fixation of cells with paraformaldehyde^36^. Herein we demonstrate that, in addition to being an efficient mitochondrial stain, MitoBlue is relocalized to lysosomes in a time-dependent manner, likely through a membrane dynamic process, and that this migration may be used as an indicator of mitochondrial recycling activity in cells subjected to different environments stimuli, or states, such as aging. Therefore, MitoBlue is much more than a simple Mitotracker, but can be used to dynamically monitor the mitochondrial quality by tracking its migration to lysosomes. We also demonstrate that MitoBlue is a suitable dye for two-photon microscopy, which allows irradiation at longer wavelengths than those required in standard fluorescence spectroscopy at 329 nm.

## Results

### Mitochondrial localization of MitoBlue

The initial mitochondrial localization of MitoBlue is consistent with the observed distribution of other delocalized lipophilic cations^37^, such as Rhodamine 123^38^, tetraphenylphosphonium^39^, and even related bisbenzamidines^40^, which suggests that MitoBlue could be electrostatically driven to the mitochondria by the large electrochemical potential generated by the electron transport chain^41^. Interestingly, unlike Rhodamine 123, MitoBlue is retained in mitochondria after fixation. Preliminary experiments using long incubation times had suggested that MitoBlue did not label mitochondria of CCCP-treated cells^38^. The mitochondrial uncoupler CCCP is a protonophore that mediates the diffusion of protons across the inner mitochondrial membrane, thus causing the loss of the membrane potential. However, repeating these experiments at shorter times and using positive controls, indicate that the mitochondria staining is relatively independent of the membrane potential. Therefore, we treated HeLa cells during 5 min with Carbonyl Cyanide 3-ChloroPhenylhydrazone (CCCP), prior to their staining with either MitoBlue, and tetramethylrhodamine ethyl ester (TMRE), or Mitotracker Deep Red (MTDR) as controls. As shown in Fig. 1A (left column), MitoBlue targeted mitochondria equally efficiently in the presence of CCCP, while mitochondrial staining with TMRE (Fig. 1A, middle column) and MTDR (Fig. 1A, right column) fainted upon incubation with the uncoupler. This experiment was repeated using FCCP as the uncoupling agent with the same results, thus confirming that MitoBlue targets mitochondria in a membrane potential-independent manner.

MitoBlue, like propamidine or 4′,6-Diamidino-2-phenylindole dihydrochloride (DAPI), belongs to the bisbenzadimine family of DNA-binding agents, and indeed, we have previously demonstrated that MitoBlue displays significant affinity for A/T-rich DNA sequences *in vitro*^36^. Therefore, we wondered whether MitoBlue might target mitochondrial DNA (mtDNA). To answer that question, HeLa cells were stained with MitoBlue and Mitotracker Deep Red (MTDR), fixed with PFA, and subjected to immunofluorescence staining using with antibodies against mtDNA. Analysis of this preparation by confocal microscopy showed the expected punctuated pattern of mtDNA concentrated in nucleolids (Fig. 1B, mtDNA)^42^; in contrast, MitoBlue was diffusely distributed in mitochondria and matching the staining pattern of MTDR, clearly indicating no mtDNA targeting. To obtain more insight into the exact mitochondrial localization of MitoBlue, COS-7 cells stained with MitoBlue were subjected to immunofluorescence analysis by simultaneously using antibodies against Tom20, a mitochondrial membrane protein^43^, and the mitochondrial matrix resident protein Pyruvate Dehydrogenase E2/E3bp (PDH). The images in Fig. 1C showed a clear difference in the staining pattern of MitoBlue and Tom20, but a clear colocalization with PDH (Fig. 1C), suggesting that MitoBlue is dominantly targeting the mitochondrial matrix and/or inner membrane. In fact, line scan analysis further shows that the peaks of fluorescence intensity of MitoBlue and PDH are between the two peaks of Tom20 (Fig. 1D).

### MitoBlue is transferred from mitochondria to lysosomes with time

**Figure 2:**
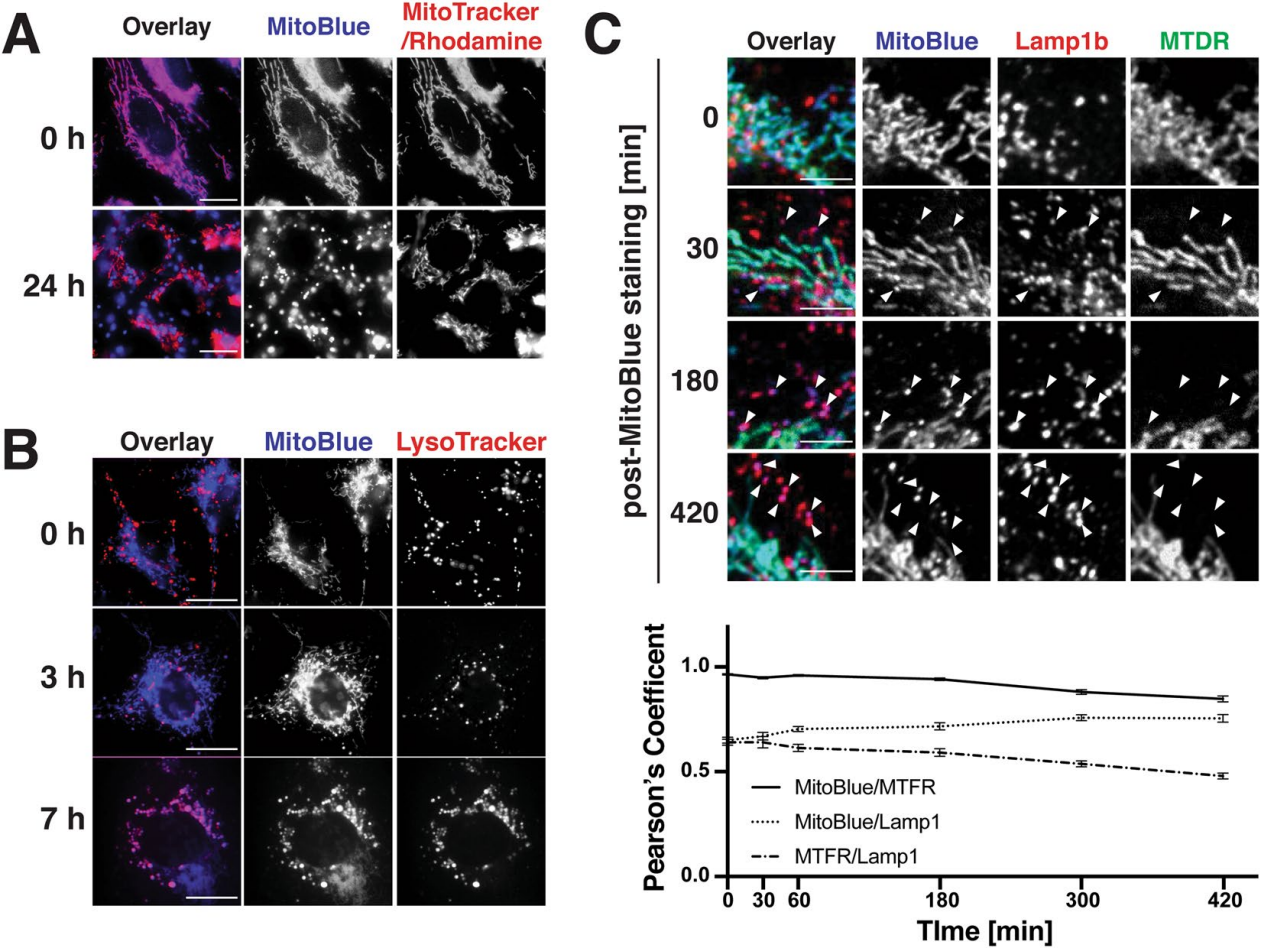
MitoBlue travels from mitochondria to lysosomes with time. (**A**) Co-staining untreated cells with Mitotracker. Vero cells were incubated with the indicated probes. Images were acquired just after simultaneous staining with Mitotracker Red and MitoBlue (0 h, top panels) or just after staining with Rhodamine 123 24 h after MitoBlue addition (24 h, bottom panels). Scale bars: 10 μm. (**B**) Co-staining untreated cells with Lysotracker. Vero cells were incubated with 5 µM MitoBlue and 50 nM Lysotracker Red. Images were acquired at indicated times after staining. (**C**) MitoBlue travels from mitochondria to lysosomes with time. HeLa cells were incubated with 5 μM MitoBlue, 100 nM Mitotracker Deep Red (MTDR) and immunostained with anti-Lamp1b antibody at the times post-staining indicated on the left of the images. Arrowheads indicate co-localization between MitoBlue and Lamp1b, but not MTDR. Scale bars: 5 μm. Line graph indicates quantification of colocalization calculated as Pearson’s coefficient between indicated labels at each time point. Error bars the means ± S.E. 17 cells were quantified at each time point.

We have previously shown that MitoBlue labels mitochondria with excellent selectivity, while causing no harm to cells^36^. To further evaluate its value as a mitochondrial stain, we wanted to assess for how long MitoBlue remains associated with mitochondria. To test this, Vero cells were incubated with 5 μM MitoBlue for 20–30 min at 37 °C, replenished with fresh medium, and then incubated at 37 °C for several hours. Observation under the microscope just after the washing step, at the beginning of the experiment, showed that MitoBlue was distributed in a filamentous pattern typical of mitochondria (Fig. 2A, 0 h, middle panel) that also matched the localization of Mitotracker Red (Fig. 2A, 0 h, right panel). At 24 h post-labelling, we counter-stained the cells with Rhodamine 123, a membrane potential-dependent dye that exclusively stains functional mitochondria. While the signal of Rhodamine 123 revealed the typical mitochondria pattern (Fig. 2A, 24 h, right panel), the signal of MitoBlue was concentrated into punctuated vesicle-like structures with a completely different distribution from that of Rhodamine 123 (Fig. 2A, 24 h, middle panel). These results suggest that with time MitoBlue leaves mitochondria and ends up accumulating in vesicle-like structures, and that mitochondria of these cells are not damaged. This behaviour is not shared neither by Mitotracker Red or Rhodamine 123, whose staining remains mitochondrial at similar incubation times (data not shown). This interesting distribution pattern led us to explore whether MitoBlue was transferred to lysosomes. As an initial approach, Vero cells were stained with MitoBlue as described before, and counter stained with Lysotracker 30 min prior to observation at selected time points. In agreement with our previous observations, MitoBlue first stains mitochondria, showing an initial distribution completely different to that of Lysotracker (Fig. 2B, 0 h, upper row); at 3 h post-staining, some vesicles were simultaneously stained with MitoBlue and Lysotracker (Fig. 2B, 3 h middle row), and at 7 h post-treatment, both reagents showed an increasing coincidence, suggesting the time-dependent transfer of MitoBlue from mitochondria to lysosomes (Fig. 2B, 7 h lower row).

To unequivocally identify the vesicles receiving the MitoBlue as lysosomes, HeLa cells were coincubated with MitoBlue and Mitotracker Deep Red (MTDR). After the fixation, cells were immunostained with antibodies against the lysosome-associated membrane glycoprotein 1 (Lamp1), a transmembrane protein which resides primarily across lysosomal membranes, and observed by confocal microscopy^44^. As shown in Fig. 2C, MitoBlue overlaps with MTDR at early times post-staining (Fig. 2C, 0 min, Pearson’s coefficient between MitoBlue and MTFR 0.964 ± 0.003, MitoBlue and Lamp1b 0.650 ± 0.014, MTFR and Lam1b 0.640 ± 0.014). The coincidence between MitoBlue and Lamp1 is already detectable at 30 min and becomes evident at later times post-staining (Fig. 2C, arrows), reinforcing our previous observation that lysosomes are the destination of the MitoBlue stain. On the other hand, MTDR labelling does not overlap at all with Lamp1, indicating that MitoBlue was selectively transferred to lysosomes (Fig. 2C, 420 min, Pearson’s coefficient between MitoBlue and MTFR 0.847 ± 0.014, MitoBlue and Lamp1b 0.754 ± 0.018, MTFR and Lam1b 0.479 ± 0.014).

### MitoBlue potentially traces several different mitochondria recycling processes

**Figure 3:**
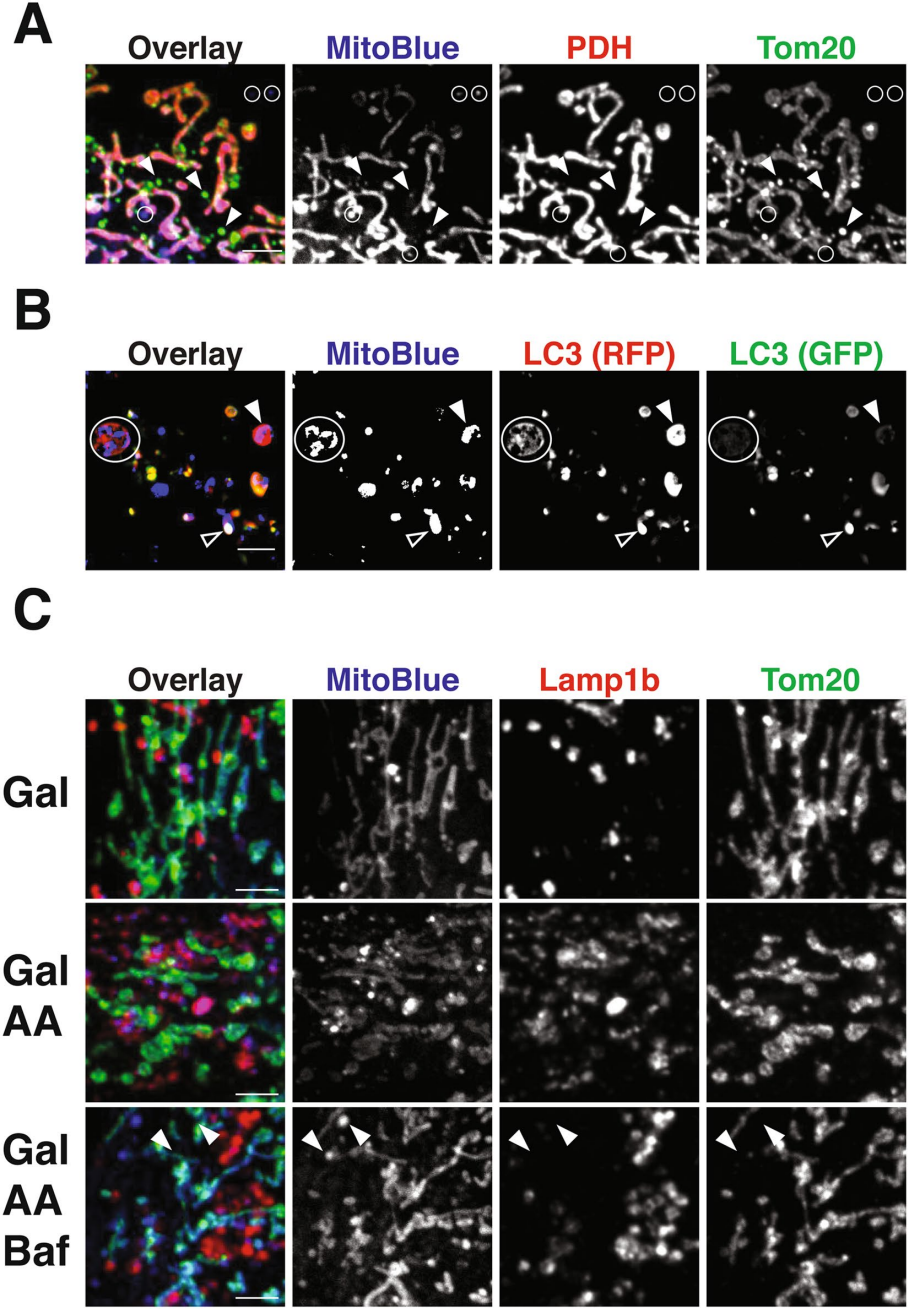
MitoBlue is transferred from mitochondria to lysosome by membrane traffic. (**A**) MitoBlue is not incorporated into conventional MDVs. COS-7 cells incubated with 5 µM MitoBlue were subjected to immunofluorescence using anti-PDH and -Tom20 antibodies. Circles show MitoBlue^+^/PDH^−^/Tom20^−^ vesicles. Scale bars: 5 μm. (**B**) Partial localization of MitoBlue with autophagic vesicles. A541 cells were transfected with ptfLC3 vector. 24 hours post-transfection, the cells were labelled with 5 µM MitoBlue for 30 minutes, washed and further incubated for 4 hours in complete medium supplemented with 1 µM Rapamycin. The fluorescence of GFP (green LC3), mRFP (red LC3) or MitoBlue was observed without fixation by confocal live-cell imaging. Arrowhead indicates vesicles simultaneously stained with MitoBlue and mRFP, open arrowhead indicates vesicles simultaneously stained with MitoBlue, GFP and mRFP and circle indicates multivesicular body containing MitoBlue. Scale bar: 5 μm. (**C**) Acidic lysosomes are required for MitoBlue transfer to lysosomes. MCH74 cells grown in galactose medium were treated incubated with 5 µM MitoBlue and 30 μM Antimycin A (AA) with or without 50 nM Bafilomycin A (BA) for 2 h. BA was added to cells 30 min prior to MitoBlue. Immunofluorescence staining was performed using anti-Tom20 antibody. Arrowheads indicate MitoBlue not co-localizing with Tom20 and Lamp1b.

Which is the mechanism of translocation of MitoBlue from mitochondria to lysosomes? The formation of MDVs (mitochondria derived vesicles) is one of the mechanisms used by nature for mitochondrial quality control, and is based on the incorporation of damaged proteins—and probably also lipids—into vesicles, which are released from mitochondria^45^. MDVs can be distinguished from fragmented mitochondria and other vesicles inside the crowded cytosol by probing with at least two mitochondrial markers. Thus, HeLa cells, post-MitoBlue staining, were subjected to immunofluorescence analysis using anti-Tom20 and PDH antibodies, which are known cargoes of MDVs. As shown in Fig. 3A, HeLa cells presented both Tom20^+^/PDH^−^ and Tom20^−^/PDH^+^ vesicle-like structures (Fig. 3A). MitoBlue also showed vesicle-like structures which were negative for either Tom20 or PDH (Fig. 3A, circles). As MitoBlue initially target mitochondria, these results suggest that it might be released from them by non-conventional MDVs.

Elimination of MitoBlue through mitophagic recycling of mitochondrial components could be another additional explanation for the unexpected behavior of the stain. To test this hypothesis, A541 cells were transfected with mRFP-GFP-LC3, which has been shown to selectively mark autophagosomes^24^, and stained with MitoBlue 24 h after transfection (Fig. 3B). The cells were then immediately observed under the confocal microscope, or further incubated for 4 h in the presence of 1 µM Rapamycin, which is known to induce both mitophagy and autophagy. As expected, immediately after incubation, MitoBlue was concentrated in the mitochondrial network, and did not superimpose with the LC3-tagged autophagic marker (not shown), but 4 h after staining and treatment with rapamycin, MitoBlue also partly colocalized with the mRFP-GFP-LC3 autophagic marker, both with GFP+/mRFP+ vesicles (Fig. 3B, empty arrow, additional examples are given under Fig. S2) and GFP^−^/mRFP^+^ vesicles (Fig. 3B, filled arrow), showing its travel through the whole mitophagic process. Importantly, MitoBlue signals are also found in GFP^−^/mRFP^+^ LC3 structures which are likely multivesicular bodies (Fig. 3B, circle), further suggesting that MitoBlue is transferred from mitochondria to lysosomes also via autophagic vesicles. On the other hand, the quick transfer to lysosomes in cells not treated with Rapamycin (Fig. 2), where mitophagy is a minor event, indicates that other transfer pathways should also be involved and that mitophagy represents a minor event in the transfer of Mitoblue from mitochondria to lysosomes.

In control galactose medium, MitoBlue showed dual localization in mitochondria and lysosomes as observed before (Fig. 3C, top panels), whereas in the presence of Antimycin A (AA), a mitochondrial complex III inhibitor that induces the formation of MDVs, MitoBlue showed a preferential accumulation in lysosomes (Fig. 3C, middle panels). Fusion between autophagosomes or late endosomes and lysosomes requires the lysosome acidification by lysosomal membrane V-ATPase. In the presence of the V-ATPase inhibitor Bafilomycin A1 (BA), MitoBlue signals accumulated in cytoplasm without colocalizing with neither MTDR nor Lamp1b (Fig. 3C, arrowheads in lower panels), supporting the idea that MitoBlue is transferred to lysosomes by a biological process(es) accompanied with membrane dynamics instead of free diffusion. In addition, incubation of Mitoblue-labelled cells with 100 µM hydroxychloroquine that inhibits mitophagy also by inhibiting the lysosome acidification did not stop the migration of the dye from mitochondria to vesicles (Fig. S1). Thus, these results also suggest that MitoBlue dynamics are associated to different pathways of mitochondrial degradation or recycling^46^.

### MitoBlue is transferred from mitochondria to lysosomes without any chemical modification

**Figure 4:**
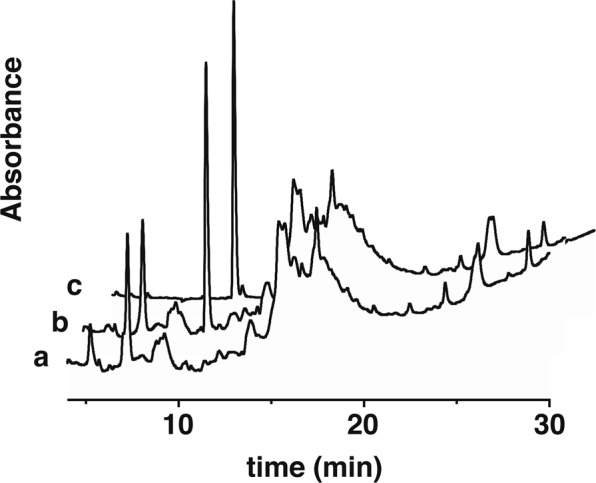
MitoBlue structure is preserved during its transfer to lysosomes. (**a**) HPLC trace of metabolic extraction of untreated Vero cells (**b**) cells incubated with MitoBlue for 9 h, (**c**) stock solution of MitoBlue as reference.

It cannot be discarded that the migration of MitoBlue from mitochondria to lysosomes might be associated to some chemical modification (perhaps oxidation). To clarify this question regarding the nature of the compound that is being targeted to the lysosomes, we incubated Vero cells with MitoBlue following the outlined protocol. After 9 h post-staining to ensure the transfer of MitoBlue to the lysosomes, cells were washed and harvested. Similarly treated cells were observed under the fluorescence microscope to assess the transference of the stain to the lysosomes (not shown). After metabolic extraction (see Methods), the pellet was analyzed by high-performance liquid chromatography (HPLC). The results in Fig. 4 show that the only difference between the material obtained from untreated cells (a) and that from MitoBlue-treated cells (b) is a single peak presenting exactly the same retention time than purified MitoBlue (c). These results are consistent with the chemical structure of MitoBlue being maintained through the whole process of mitochondrial staining and subsequent transfer to the lysosomes.

### Studying the dynamics of mitochondrial recycling with MitoBlue

**Figure 5:**
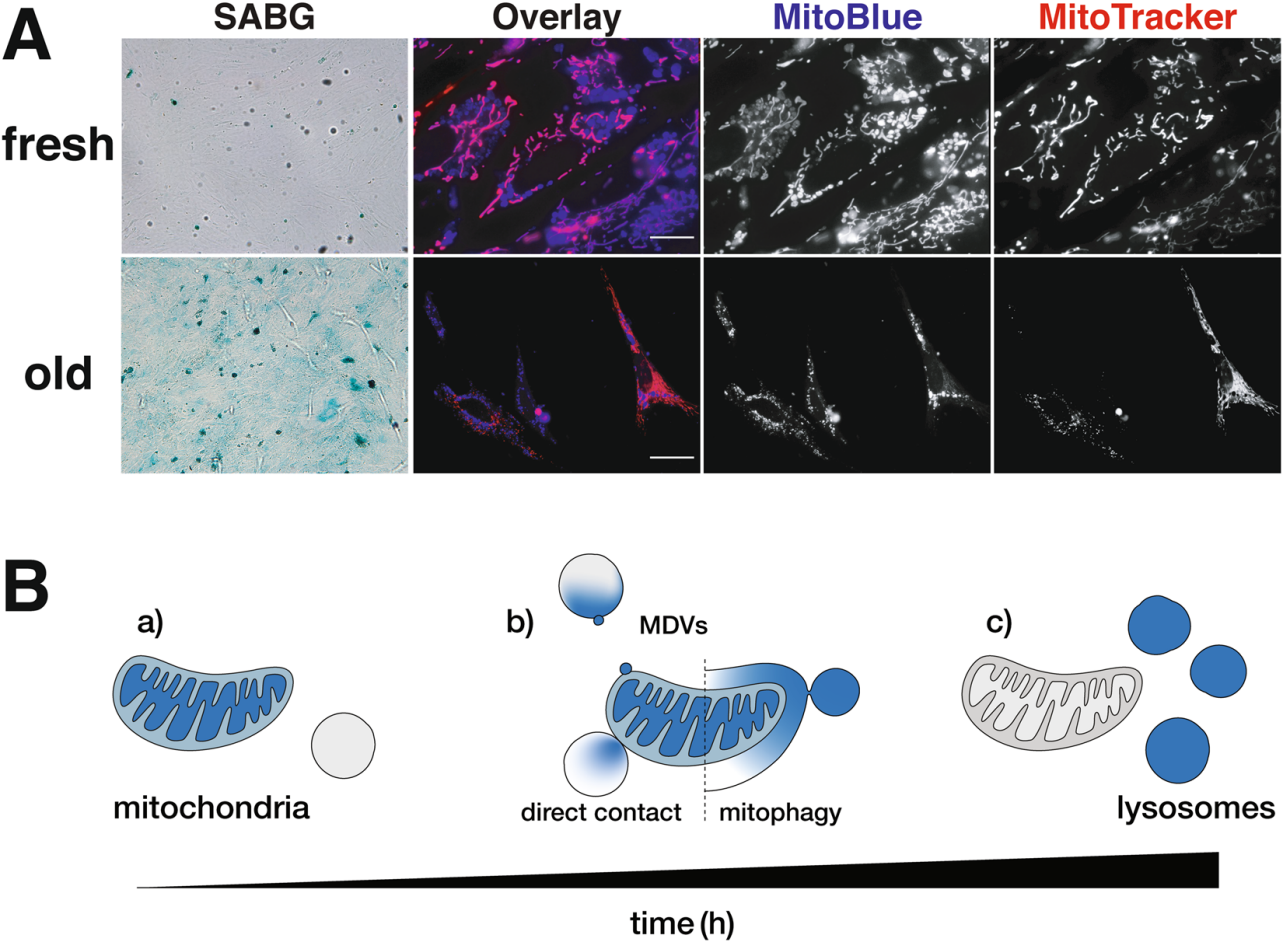
MitoBlue labels the steps in the mitochondrial life cycle, from functional mitochondria to the final lysosomal degradation. (**A**) Turnover of MitoBlue with culture age. Freshly harvested (fresh) and aged primary cultures of CEF cells (old) were stained with 5 µM MitoBlue, incubated for 3 h and then co-stained with Mitotracker Red. Identical samples were stained with X-Gal (SABG). Scale bars: 100 μm for brightfield images and 10 μm for the fluorescent pictures. (**B**) Schematic model to explain the behavior of MitoBlue in cells. a) MitoBlue firstly targets mitochondria. No lysosomal staining is observed; b) MitoBlue travels from mitochondria to lysosomes by MDVs, mitophagy and/or direct contact; c) MitoBlue dominantly labels lysosomes.

The above data suggest that MitoBlue could be used to monitor the fate of mitochondrial contents. Thus, we compared the rates of transfer of MitoBlue to lysosomes between freshly prepared CEF cells, or CEF that were kept in culture for 10 days to induce culture-induced senescence. The presence of increased β-Galactosidase activity in the ten days-old cultured CEF cells clearly indicated their senescence status (Fig. 5A, SABG column). As can be seen in Fig. 5A, at 1.5 h post-MitoBlue treatment, freshly harvested CEF showed a significant overlap between MitoBlue and Mitotracker Red staining (Fig. 5A, top row). On the other hand, older CEFs displayed a completely remodeled MitoBlue pattern, equivalent to what is observed in the fresh culture after longer post-MitoBlue staining (Fig. 5A, bottom row), suggesting a higher mitochondria recycling rate.

### MitoBlue is suitable for Two-photon microscopy

The ability of MitoBlue to act as a general marker for mitochondria is somewhat compromised by its relatively short excitation wavelength (λ_exc_ 329 nm). Thus, as a possible improvement for this dye, we decided to carry put a preliminary exploration of the performance of MitoBlue as a fluorophore in two-photon fluorescence excitation microscopy (TPM). TPM is based on the unique characteristics of the two-photon absorption process occurring in probes when excited at long excitation wavelength (700 to 1000 nm). The technique can enable the visualization of intracellular organelles of living specimens with advantages over conventional fluorescence microscopy such as increased light penetration depth, lower auto-fluorescence levels, reduced phototoxicity and photobleaching, as well as the possibility of three-dimensional imaging of living tissues and longer observation times^47,48^.

**Figure 6:**
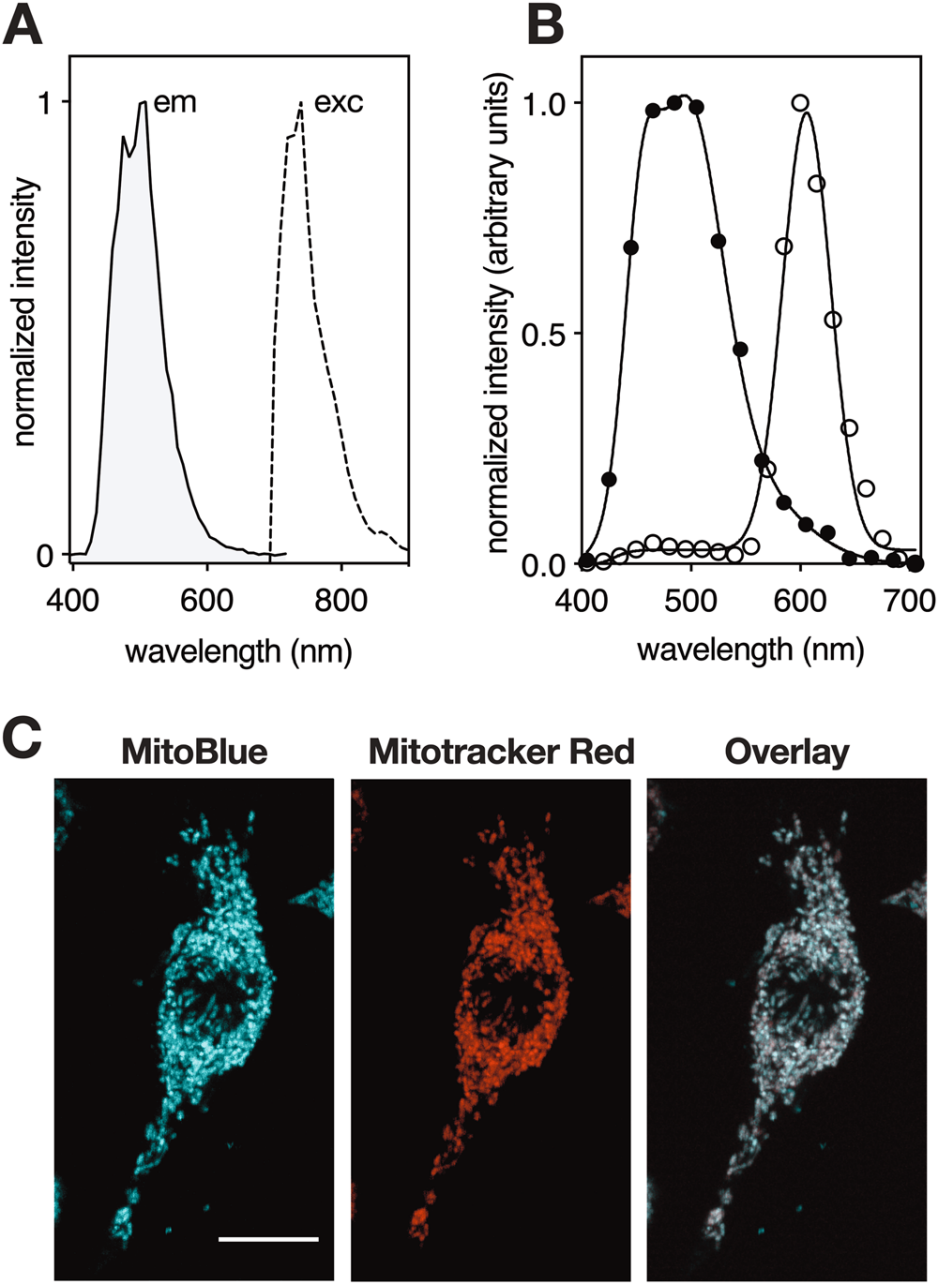
Availability of MitoBlue in TPE. (**A**) Fluorescence spectrum (λ_exc_ = 740 nm, solid line), and excitation spectrum (λ_em_ = 740 nm, dashed line) of MitoBlue under TPE conditions. **(B)** Fluorescence spectra recorded inside the cell of MitoBlue (∙) and Mitotracker Red (○), λ_exc_ = 740 nm TPE conditions. **(C**) N13 cells incubated with 5 µM MitoBlue and 50 nM Mitotracker Red, two-photon excitation microscopy images recorded at λ_exc_ = 740 nm, with the emission channel at 480 nm for MitoBlue and 600 nm for Mitotracker Red. The white bar corresponds to 10 μm.

To confirm the potential of MitoBlue for TPM, we tested its luminescent response in water, under two-photon excitation conditions using a 740 nm excitation wavelength (Fig. 6A left). As expected, the compound showed an emission spectrum maximum at c.a. 500 nm. The two photon absorption (TPA) cross-sections were determined using the Two Photon Excited Fluorescence (TPEF) method, assuming that the quantum efficiencies after two-photon excitation are the same as those after one-photon excitation^49,50^. TPA cross-sections were obtained by calibration against Rhodamine B with a known δ value in MeOH solution, and calculated on the basis of the following expression:

$${\delta }_{S}={\delta }_{R}\frac{{C}_{R}{\eta }_{R}{\phi }_{R}}{{C}_{S}{\eta }_{S}{\phi }_{S}}\frac{{F}_{s}}{{F}_{R}}$$

where δ is the TPA cross-section, C and η are the concentration and refractive index of the sample solution, and F is the integrated area under the TPEF spectrum. MitoBlue shows TPA cross-sections δ values of 6.0 GM [1 GM (Goeppert–Mayer unit) = 10^−50^ cm^4^ s molecule^–1^ photon^−1^] at 740 nm. The two photonic nature of the observed phenomena was confirmed by the quadratic dependence of the emission on the laser power.

To test the practical applicability of MitoBlue for cell imaging, cultured N13 mouse microglia cells were incubated with 5 µM MitoBlue and 50 nM Mitotracker for 45 min under standard growth conditions. Cells were washed with fresh growth medium and examined directly by TPM, observing the characteristic blue emission of the compound that, as expected, colocalized with that of Mitotracker Red using the same 740 nm two-photon excitation (TPE) wavelength (Fig. 6C)^51^.

## Discussion

MitoBlue is a synthetic bisbenzamidine derivative that stains mitochondria with high specificity through a still undetermined mechanism. The polarization of the mitochondrial membrane does not appear to be the main driving force directing MitoBlue to the mitochondria, indicating that other physicochemical effects or even specific interactions at the mitochondrial compartment are responsible such intracellular selectivity and staining. Thus, prolonged incubation with depolarizing agents seems to reduce the amount of MitoBlue reaching mitochondria, but short incubation times with these agents, that typically block other dyes, do not show any appreciable effect on the localization of MitoBlue. Although MitoBlue has a delocalized positive charge typical of mitochondrial dyes, MitoBlue is retained after fixation and, more importantly, transferred through membrane traffic processes to lysosomes through different pathways, a characteristic not shared with other available mitochondrial stains. Thus, these data suggest that there should be some interaction between MitoBlue and specific mitochondrial components that retains this stain in the mitochondria. We have previously demonstrated the ability of MitoBlue to specifically bind to DNA *in vitro*, so our initial hypothesis was that mitochondrial DNA might anchor our stain to the compartment. However, we show in this manuscript that there is no association between MitoBlue and mitochondrial DNA in living cells, therefore ruling out that possibility (Fig. 1).

We found that MitoBlue localizes with PDH and not with Tom20, which strongly suggests its association with the inner mitochondrial membrane (IMM). Cardiolipin (CL) is the most characteristic of the components of the IMM and amounts to up to 20% of its total lipid composition. Its large head group and its cone-shaped structure are unique between membrane lipids, and positively charged guanidinium and amidinium groups such as those present in MitoBlue are known to strongly interact with anionic species, including carboxylates and phosphates^52,53^, thus providing a strong supramolecular rationale for the interaction between MitoBlue and CL. Furthermore, cationic aminoglycosides structurally resembling MitoBlue, such as 3,6-dinonyl neamine, have been shown to bind cardiolipin in cells^54^; CL is also present in the membranes of bacteria and mycoplasms, and both organisms are also targeted by MitoBlue (unpublished results). Altogether, these observations pont to CL as a likely responsible for the selective capture of MitoBlue in the IMM.

After reaching and staining mitochondria, MitoBlue is gradually transported out of this organelle, ending up in lysosomes. Such migration is not due to a chemical modification that might alter the affinity of the stain for the mitochondria (Fig. 5). There are currently four known pathways that enable mitochondrial quality control^45^. Two of them are specifically dedicated to degrade mitochondrial proteins: specific proteases in the mitochondrial matrix and intermembrane space degrade damaged proteins^55^, while some mitochondrial proteins at the outer membrane are degraded at the proteasome following ubiquitination^56^. On the other hand, dysfunctional mitochondria can be removed by their targeting to autophagosomes, through a mechanism called mitophagy, for whole organelle elimination^49,57^. Dysfunctional mitochondrial components can finally be incorporated into MDVs, vesicles that are ultimately targeted to lysosomes for recycling; this pathway not only takes care of protein recycling, but also processes membrane lipids and possibly other materials^58,59^. The characterization of these pathways relies on the use of tagged proteins or specific antibodies that target just a few of the more than 1000 proteins that are present in the mitochondria. More importantly, the latter two pathways need membrane dynamics and engage other membrane compartments.

In our study, we have explored the evolution of MitoBlue in both LC3-positive and negative structures (Fig. 3B), and showed that MitoBlue is incorporated into both mitophagic and MDVs pathways. Taking account of the low frequency of MDVs and mitophagy in untreated cells, direct contact is another possible mechanism for MitoBlue transfer from mitochondria to lysosomes^49,60^. These two organelles are highly dynamic, and form contact sites with other membrane compartments. In recent years, direct contact between mitochondria and lysosomes (multivesicular body as well) have been reported, including “kiss and run” events, where ions and lipids are transferred^61^. Some of such contacts could be mediating recycling processes by direct material transfer that might not be detected unless they include typically monitored mitochondrial proteins. Membrane contact sites can be evaluated with TEM or fluorescence microscope measuring the distance between membranes, or calculating the co-localization coefficient such as Mander’s and Pearson’s. Functional evaluation however, relies on probing materials that can be transferred between organelles and followed either by imaging analysis using florescence probes or biochemical analysis, such as pulse-chase using radio isotopes. Between both methods, imaging analysis has the advantage of ensuring the direct localization of the probe. Because most probes used in these assays target organelles that are either the origin or the destination, they are not useful to directly analyze the transfer process. For example, calcium ions are transferred between endoplasmic reticulum and mitochondria. Since mitochondrial calcium uniporter possesses low affinity to calcium ions, membrane contact sites are required to generate a hot spot where calcium ions are concentrated high enough to be uptaken by mitochondria^62,63^.

In contrast, MitoBlue firstly targets mitochondria, then travels to lysosomes in various cell types and at different vital status. As the first target is not dependent on the mitochondrial status, MitoBlue is able to trace the dynamic processes. Transfer of MitoBlue from mitochondria to lysosomes is accelerated under incubation with hydrogen peroxide and in aged cells, where mitochondrial quality control may be activated. Therefore, MitoBlue can be used to easily monitor the changes in mitochondrial quality control mechanisms in different cells and cellular conditions. MitoBlue staining pattern is unique among mitochondrial stains and recombinant fluorescent probes, allowing to monitor in a non-invasive dynamic fashion the mitochondrial recycling mechanism in a variety of situations in living cells. MitoBlue represents a unique tool for the study of these processes that will allow the detailed characterization of communication between mitochondria and lysosomes.

## Materials and Methods

### Chemicals and antibodies

MitoBlue was synthesized as reported previously reported^64^. DMSO, carbonyl cyanide 3-chlorophenylhydrazone, antimycin A, bafilomycin A were purchased from *Sigma*. Mitotracker Red, Mitotracker Deep Red, and tetramethylrhodamine ethyl ester were purchased form *Thermo Fisher*. Rabbit polycolonal anti-Tom 20 antibody (# Sc-11415) and mouse monoclonal anti-Lamp1b antibody (# Sc-17768) were from *Santa Cruz Biotecnology Inc*., mouse monoclonal PDH antibody was from *Abcam* (# ab110333) and mouse monoclonal anti-DNA antibody was from *Progen Biotechnik* (# 6104). Anti-mouse and anti-rabbit conjugated secondary antibodies were all obtained from *Sigma*.

### Standard labelling protocol for MitoBlue and other organulle-specific stains

Monolayers of the corresponding cell line were incubated with 5 μM MitoBlue in DMEM containing no additives, for 20–30 min at 37 °C. The cells were then washed 3 times with pre-warmed DMEM and finally overlayed with fresh DMEM containing 10% FBS and incubated for the times indicated in each experiment.

### Cell cultures

Specific-pathogen-free (SPF) 9-day old embryonated eggs were generously donated by MSN. Primary cultures of chicken embryo fibroblasts (CEF) were prepared from the chicken 9-day old embryos as described^65^, and grown in monolayers in medium 199 supplemented with 10% (w/v) tryptose-phosphate broth and 5% (v/v) calf serum. The study protocol is exempt from the need for ethical approval under Spanish law (R.D. 53/2013, Law 32/2007). COS-7, Vero and HeLa cells obtained from ATCC and MCH64 cells obtained from Montreal Children’s Hospital were grown in monolayers in medium D-MEM supplemented with 10% fetal bovine serum. For oxidative stress-induced MDVs experiments, cells were pre-treated with 50 nM of bafilomycin A1 for 30 min, then MitoBlue was added to cells. Culture medium was replaced with galactose medium supplemented with or without 30 mM of antimycin A and 50 nM of bafilomycin A1. After 3 h incubation, cells were fixed and subjected to immunofluorescence staining.

### Multiphoton microscopy

The two-photon absorption cross-section were determined on a Leica SP5 AOBS MP instrument according to standard procedures and within a laser power regime where the fluorescence was proportional to the square of the laser excitation power. In this way it was ensures that only two-photon absorption occurred.

Samples were analysed using an inverted Leica SP5 AOBS MP confocal microscope equipped with a MaiTai Ti:Sapphire HP laser (Spectra-Physics, INc.) tunable between 690 and 1040 nm. The imaging was performed by using a 63xPLAN APO oil immersion objective (NA 1.4). The emission and excitation spectra data were registered with the Leica LAS AF software.

For testing the practical applicability of MitoBlue in cellular settings. N13 cells were incubated with 5 µM MitoBlue for 45 min prior to imaging.4 Live cells were observed in the microscope under optimal conditions in 8-well glass-bottomed slides at 37 C and 5% CO2. Using TP excitation at 740 nm the characteristic blue emission of the compound could easily be detected using minimal power (<1% of max laser power) detecting emissions from 500–500 nm. To evaluate the localization of the dye, cells were co-stained with 2 µM of the mitochondrial marker Mitotracker Red CMX-ROS (Thermofisher). The ability of this marker to work under TPE conditions allowed the simultaneous visualization of both dyes.5 Fig. 2 shows TP microscopy images of N13 cells incubated with 5 µM MitoBlue and 500 nM Mitotracker red with TP excitation at 740 nm and emissions detected at 500–500 nm and 565–605 nm, respectively. Transmitted brightfield images were captured separately using a 405 nm laser. The emission spectra of both dyes recorded inside the cell, using 740 nm of excitation wavelength is also shown.

As we can observed, MitoBlue is co-localized with the mitochondrial marker, and can be easily visualized under TPM.

### Transfections and IF microscopy

Plasmid ptfLC3 was a gift from Tamotsu Yoshimori (Obtained from *Addgene*, plasmid # 21074)^66^. Transfections of cell monolayers were done with the Lipofectamine Plus reagent (*Invitrogen*), according to the manufacturer’s instructions. Transfected cells were incubated at 37 °C for 18 to 24 h, unless otherwise stated. For immunofluorescence studies, the cells were permeabilized by incubation with 0.1% Triton X-100/PBS for 10 min at room temperature followed by incubation with 5% BSA/PBS for 10 min. The primary antibodies indicated for any particular experiment were added to cell monolayers in 5% FBS/PBS and incubated for 2 h at rt. After washing the monolayers three times with 5% FBS/PBS, cells were incubated for 1 h with secondary antibodies. Images were alternatively obtained with an *Olympus* DP-71 digital camera mounted on an *Olympus* BX51 fluorescence microscope, or with an *Olympus* IX70 equipped with a TillPhotonics camera. Confocal images were acquired either with an *Olympus* FV1000 confocal microscope or with an *Andor Dragonfly* spinning disk confocal system mounted on a *Nikon* TiE microscope equipped with a Zyla 4.2 PLUS camera (*Andor*). Colocalization was quantified as Pearson’s coefficient using JACoP, *ImageJ* plugin^67^.

### Metabolic extraction of MitoBlue from stained cells

In order to confirm the stability and integrity of MitoBlue inside the cell, we incubated MitoBlue 20 µM for 45 min in a cell culture dish; the supernatant was discarded and cells were rinsed with PBS and kept for 9 h in DMEM with FBS. The supernatant was discarded once more, the cells were rinsed with PBS and finally removed and lysated^68–70^.

### Senescence-associated β galactosidase staining

Cultured cells were washed 3 times with PBS, fixed with 4% paraformaldehyde, permeabilized with 0.5% Triton X-100 in PBS and then overlayed with staining solution (1 mg/mL 5-bromo-4-chloro-3-indolyl-β-d-galactopyranoside, citric acid/sodium phosphate buffer pH 6.0, 5 mM potassium ferricyanide, 5 *mM* potassium ferrocyanide, 150 mM NaCl, and 2 mM MgCl_2_. After 2 h at 37 °C the staining solution was removed, the cells washed twice with PBS and mounted for microscopy.

## Supplementary information

Supplementary Information.

## References

[1] Otera H, Mihara K (2011). Molecular mechanisms and physiologic functions of mitochondrial dynamics. J. Biochem..

[2] May AI, Devenish RJ, Prescott M (2012). The many faces of mitochondrial autophagy: making sense of contrasting observations in recent research. Int. J. Cell Biol..

[3] Levine B, Kroemer G (2008). Autophagy in the pathogenesis of disease. Cell.

[4] Shutt TE, McBride HM (2013). Staying cool in difficult times: mitochondrial dynamics, quality control and the stress response. Biochim. Biophys. Acta.

[5] Mijaljica D, Prescott M, Devenish RJ (2007). Different fates of mitochondria: alternative ways for degradation?. Autophagy.

[6] Ashrafi G, Schwarz TL (2013). The pathways of mitophagy for quality control and clearance of mitochondria. Cell Death Differ..

[7] Nunnari J, Suomalainen A (2012). Mitochondria: in sickness and in health. Cell.

[8] Itoh K, Nakamura K, Iijima M, Sesaki H (2013). Mitochondrial dynamics in neurodegeneration. Trends Cell Biol..

[9] Janku F, McConkey DJ, Hong DS, Kurzrock R (2011). Autophagy as a target for anticancer therapy. Nat. Rev. Clin. Oncol..

[10] Schriner SE (2005). Extension of murine life span by overexpression of catalase targeted to mitochondria. Science.

[11] Madeo F, Tavernarakis N, Kroemer G (2010). Can autophagy promote longevity?. Nat. Cell Biol..

[12] Azzouz M (2004). VEGF delivery with retrogradely transported lentivector prolongs survival in a mouse ALS model. Nature.

[13] Kuma A (2004). The role of autophagy during the early neonatal starvation period. Nature.

[14] Yousif LF, Stewart KM, Kelley SO (2009). Targeting mitochondria with organelle-specific compounds: strategies and applications. Chembiochem.

[15] Poot M (1996). Analysis of mitochondrial morphology and function with novel fixable fluorescent stains. J. Histochem. Cytochem..

[16] Mathur A, Hong Y, Kemp BK, Barrientos AA, Erusalimsky JD (2000). Evaluation of fluorescent dyes for the detection of mitochondrial membrane potential changes in cultured cardiomyocytes. Cardiovasc. Res..

[17] Mizushima N, Yoshimori T, Levine B (2010). Methods in mammalian autophagy research. Cell.

[18] Rosado CJ, Mijaljica D, Hatzinisiriou I, Prescott M, Devenish RJ (2008). Rosella: a fluorescent pH-biosensor for reporting vacuolar turnover of cytosol and organelles in yeast. Autophagy.

[19] Sargsyan A (2015). Rapid parallel measurements of macroautophagy and mitophagy in mammalian cells using a single fluorescent biosensor. Sci. Rep..

[20] Zhang J, Campbell RE, Ting AY, Tsien RY (2002). Creating new fluorescent probes for cell biology. Nat. Rev. Mol. Cell Biol..

[21] Fernández-Suárez M, Ting AY (2008). Fluorescent probes for super-resolution imaging in living cells. Nat. Rev. Mol. Cell Biol..

[22] Giepmans BNG, Adams SR, Ellisman MH, Tsien RY (2006). The fluorescent toolbox for assessing protein location and function. Science.

[23] Kabeya Y (2000). LC3, a mammalian homologue of yeast Apg8p, is localized in autophagosome membranes after processing. EMBO J..

[24] Kimura S, Noda T, Yoshimori T (2007). Dissection of the autophagosome maturation process by a novel reporter protein, tandem fluorescent-tagged LC3. Autophagy.

[25] Jensen EC (2012). Use of fluorescent probes: their effect on cell biology and limitations. Anat. Rec..

[26] Lavis LD, Raines RT (2008). Bright ideas for chemical biology. ACS Chem. Biol..

[27] Minamikawa T (1999). Chloromethyl-X-rosamine (MitoTracker Red) photosensitises mitochondria and induces apoptosis in intact human cells. J. Cell Sci..

[28] Pendergrass W, Wolf N, Poot M (2004). Efficacy of MitoTracker GreenTM and CMXrosamine to measure changes in mitochondrial membrane potentials in living cells and tissues. Cytometry.

[29] Guo D (2011). Cell-Permeable Iminocoumarine-Based Fluorescent Dyes for Mitochondria. Org. Lett..

[30] Neto BAD (2011). Synthesis, properties and highly selective mitochondria staining with novel, stable and superior benzothiadiazole fluorescent probes. RSC Adv..

[31] Leung CWT (2013). A photostable AIE luminogen for specific mitochondrial imaging and tracking. J. Am. Chem. Soc..

[32] Yu G (2016). A pillar[5]arene-based [2]rotaxane lights up mitochondria. Chem. Sci..

[33] Shin WS (2016). Mitochondria-targeted aggregation induced emission theranostics: crucial importance of *in situ* activation. Chem. Sci..

[34] Neto BAD, Corrêa JR, Silva RG (2013). Selective mitochondrial staining with small fluorescent probes: importance, design, synthesis, challenges and trends for new markers. RSC Adv..

[35] Liu Y (2016). A Cyanine Dye to Probe Mitophagy: Simultaneous Detection of Mitochondria and Autolysosomes in Live Cells. J. Am. Chem. Soc..

[36] Sánchez MI, Martínez-Costas J, Mascareñas JL, Vázquez ME (2014). MitoBlue: a nontoxic and photostable blue-emitting dye that selectively labels functional mitochondria. ACS Chem. Biol..

[37] Trapp S, Horobin RW (2005). A predictive model for the selective accumulation of chemicals in tumor cells. Eur. Biophys. J..

[38] Johnson LV, Walsh ML, Chen LB (1980). Localization of mitochondria in living cells with rhodamine 123. Proc. Natl. Acad. Sci. USA.

[39] Ross MF (2005). Lipophilic triphenylphosphonium cations as tools in mitochondrial bioenergetics and free radical biology. Biochemistry.

[40] Lansiaux A (2002). Distribution of furamidine analogues in tumor cells: targeting of the nucleus or mitochondria depending on the amidine substitution. Cancer Res..

[41] Ross MF (2006). Accumulation of lipophilic dications by mitochondria and cells. Biochem. J.

[42] Brown TA (2011). Superresolution fluorescence imaging of mitochondrial nucleoids reveals their spatial range, limits, and membrane interaction. Mol. Cell. Biol..

[43] Jakobs S, Wurm CA (2014). Super-resolution microscopy of mitochondria. Curr. Opin. Chem. Biol..

[44] Cheng X-T (2018). Characterization of LAMP1-labeled nondegradative lysosomal and endocytic compartments in neurons. J. Cell Biol..

[45] Sugiura A, McLelland G-L, Fon EA, McBride HM (2014). A new pathway for mitochondrial quality control: mitochondrial-derived vesicles. EMBO J..

[46] Todkar K, Ilamathi HS, Germain M (2017). Mitochondria and Lysosomes: Discovering Bonds. Front Cell Dev Biol.

[47] Helmchen F, Denk W (2005). Deep tissue two-photon microscopy. Nat. Methods.

[48] Denk W, Strickler JH, Webb WW (1990). Two-photon laser scanning fluorescence microscopy. Science.

[49] Collado D (2014). Energy transfer in aminonaphthalimide-boron-dipyrromethene (BODIPY) dyads upon one- and two-photon excitation: applications for cellular imaging. Chem. Asian J..

[50] Terenziani F, Katan C, Badaeva E, Tretiak S, Blanchard-Desce M (2008). Enhanced Two-Photon Absorption of Organic Chromophores: Theoretical and Experimental Assessments. Adv. Mater..

[51] Miao F (2014). Novel fluorescent probes for highly selective two-photon imaging of mitochondria in living cells. Biosens. Bioelectron..

[52] Yamada H, Wu Z-Q, Furusho Y, Yashima E (2012). J. Am. Chem. Soc..

[53] Neal JF (2019). Interfacial Supramolecular Structures of Amphiphilic Receptors Drive Aqueous Phosphate Recognition. J. Am. Chem. Soc..

[54] Sautrey G (2016). Negatively Charged Lipids as a Potential Target for New Amphiphilic Aminoglycoside Antibiotics: A Biophysical Study. J. Biol. Chem..

[55] Tatsuta T, Langer T (2009). AAA proteases in mitochondria: diverse functions of membrane-bound proteolytic machines. Res. Microbiol..

[56] Xu S, Peng G, Wang Y, Fang S, Karbowski M (2011). The AAA-ATPase p97 is essential for outer mitochondrial membrane protein turnover. Mol. Biol. Cell.

[57] Youle RJ, Narendra DP (2011). Mechanisms of mitophagy. Nat. Rev. Mol. Cell Biol..

[58] Soubannier V (2012). A vesicular transport pathway shuttles cargo from mitochondria to lysosomes. Curr. Biol..

[59] Neuspiel M (2008). Cargo-selected transport from the mitochondria to peroxisomes is mediated by vesicular carriers. Curr. Biol..

[60] Wong YC, Ysselstein D, Krainc D (2018). Mitochondria-lysosome contacts regulate mitochondrial fission via RAB7 GTP hydrolysis. Nature.

[61] Liu X, Weaver D, Shirihai O, Hajnóczky G (2009). Mitochondrial “kiss-and-run”: interplay between mitochondrial motility and fusion-fission dynamics. EMBO J..

[62] Hayashi T, Rizzuto R, Hajnoczky G, Su T-P (2009). MAM: more than just a housekeeper. Trends Cell Biol..

[63] Baughman JM (2011). Integrative genomics identifies MCU as an essential component of the mitochondrial calcium uniporter. Nature.

[64] Sánchez MI, Martínez-Costas J, Mascareñas JL, Vázquez ME (2014). MitoBlue: a nontoxic and photostable blue-emitting dye that selectively labels functional mitochondria. ACS Chem. Biol..

[65] Martinez-Costas J, Varela R, Benavente J (1995). Endogenous enzymatic activities of the avian reovirus S1133: identification of the viral capping enzyme. Virology.

[66] Kimura S, Noda T, Yoshimori T (2007). Dissection of the autophagosome maturation process by a novel reporter protein, tandem fluorescent-tagged LC3. Autophagy.

[67] Bolte S, Cordelières FP (2006). A guided tour into subcellular colocalization analysis in light microscopy. J. Microsc..

[68] Sheikh KD, Khanna S, Byers SW, Fornace A, Cheema AK (2011). Small molecule metabolite extraction strategy for improving LC/MS detection of cancer cell metabolome. J. Biomol. Tech..

[69] Masson P, Alves AC, Ebbels TMD, Nicholson JK, Want EJ (2010). Optimization and evaluation of metabolite extraction protocols for untargeted metabolic profiling of liver samples by UPLC-MS. Anal. Chem..

[70] Sapcariu SC (2014). Simultaneous extraction of proteins and metabolites from cells in culture. MethodsX.

