# Supplementary material for: MitoBlue as a tool to analyze the mitochondria-lysosome communication
Source: Sci Rep. 2020 Feb 26;10:3528. doi: 10.1038/s41598-020-60573-7 (PMC7044336; doi:10.1038/s41598-020-60573-7)
Supplement: Supplementary file 1 — Supplementary Information. [file 41598_2020_60573_MOESM1_ESM.pdf]

Supplementary Information

MitoBlue as a tool to analyze the mitochondria-lysosome communication

Mateo I. Sánchez, Yolanda Vida, Ezequiel Pérez-Inestrosa, José L. Mascareñas, M. Eugenio Vázquez, Ayumu Sugiura, and José Martínez-Costas

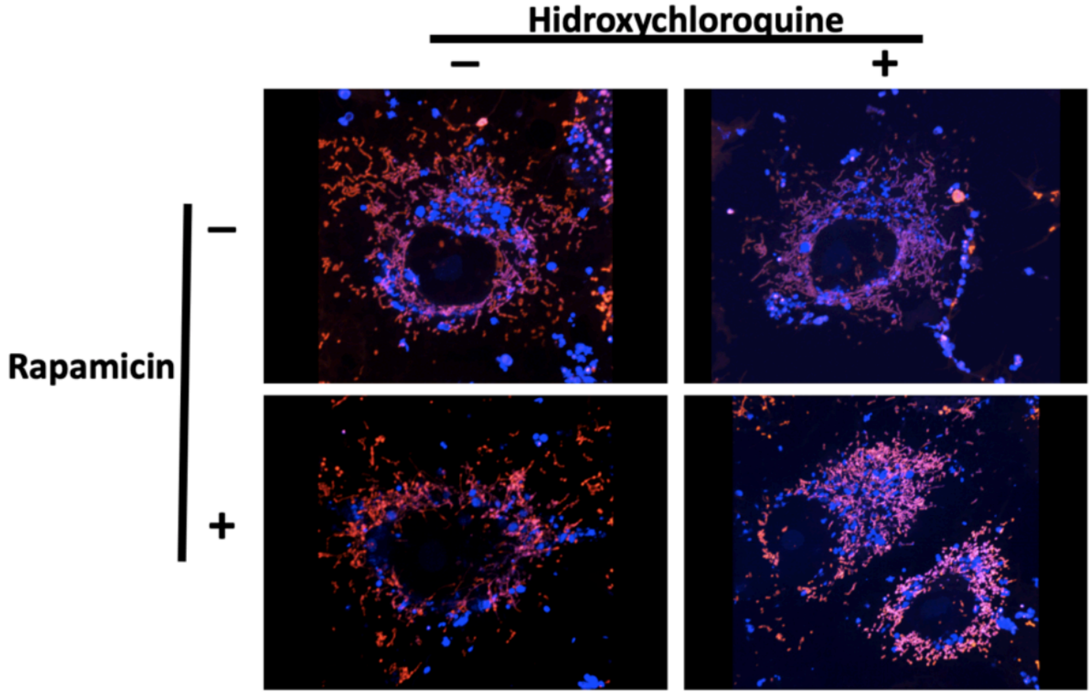

Figure S1- Mitoblue is transferred from the mitochondria to vesicles in the presence of Hidroxychloroquine. Vero cells were grown on glass-bottom multiwell plates in DMEM containing 10% FBS. Cells were washed with PBS three times, and incubated for 30 minutes with 5µM of Mitoblue in DMEM in the absence of serum. Then, the cells were washed three times with PBS, overlayed with DMEM containing 10% FBS and further incubated at 37ºC in a CO_2 incubator. In the samples indicated on the figure, the incubation medium additionally contained 1 µM of rapamycin or 100 µM hydroxyquinoline. After 6 hours, all samples were labelled with Mitotracker Red for 20 minutes and observed without fixation on a Dragonfly spinning disk confocal apparatus (Andor) mounted on a Nikon TiE microscope. Images were processed with the Imaris software. Maximum intensity projections are shown. We observed no difference in the

degree of migration of Mitoblue to round vesicles in the presence of hydroxychoroquine, even after rapamycin treatment.

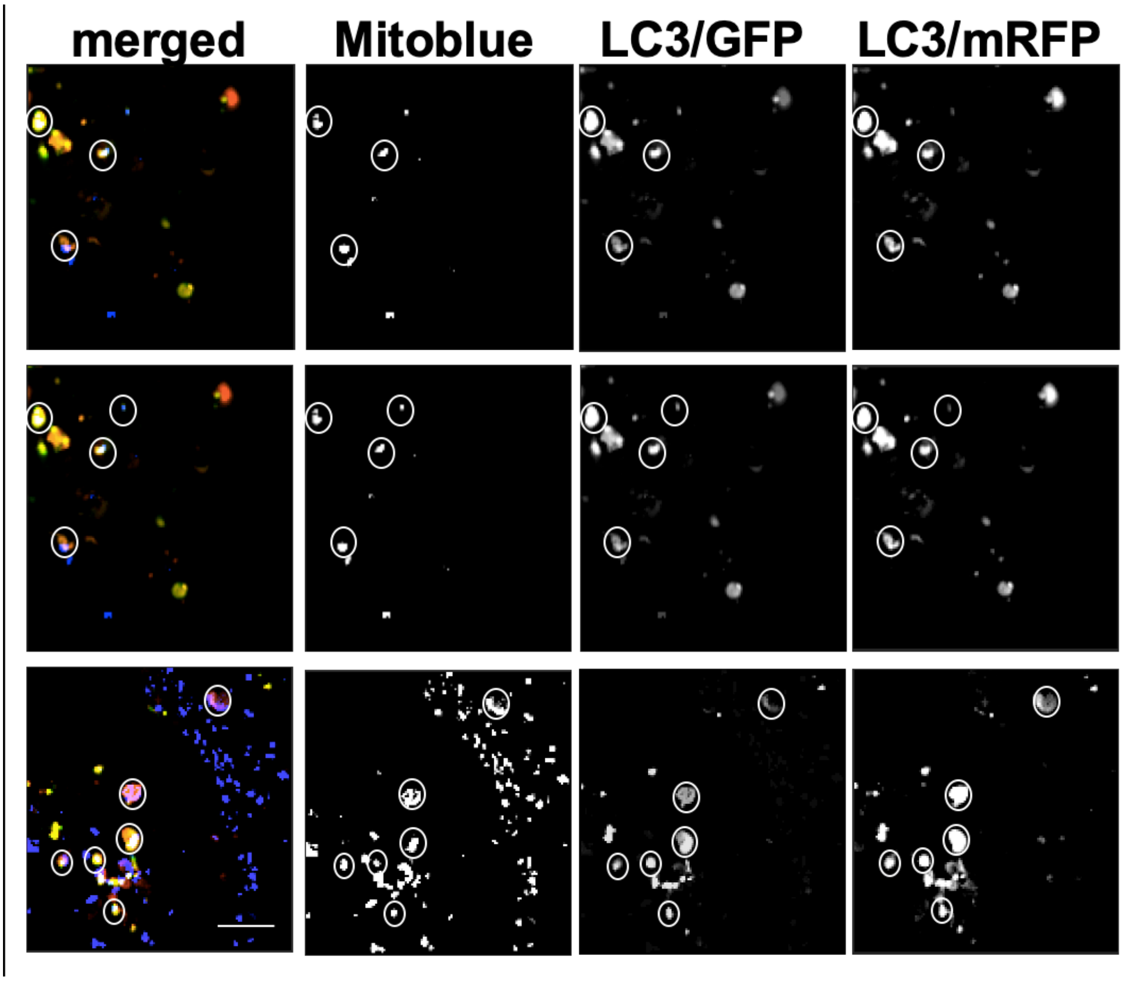

Figure S2- Co-localization of Mitoblue with LC3-containing vesicles. The figure shows confocal deconvoluted images obtained from cells processed exactly as described in figure 3 B of the main manuscript. Briefly, A541 cells were transfected with ptfLC3 vector. 24 hours post-transfection, the cells were labelled with 5 µM MitoBlue for 30 minutes, washed and further incubated for 4 hours in complete medium supplemented with 1µM Rapamycin. The fluorescence of GFP (green LC3), mRFP (red LC3) or MitoBlue was observed without fixation by confocal live-cell imaging using a Dragonfly spinning disk confocal system (Andor) mounted on a Nikon TiE microscope. Images were processed with the Imaris software. Open circles show the co-localization of some vesicles containing Mitoblue that was emitted from mitochondria, with LC3-labelled vesicles that were not fused with lysosomes as shown by their green fluorescence.
